# Energy Drink Consumption Among Finnish Adolescents: Prevalence, Associated Background Factors, Individual Resources, and Family Factors

**DOI:** 10.3389/ijph.2021.620268

**Published:** 2021-05-07

**Authors:** Maija Puupponen, Jorma Tynjälä, Asko Tolvanen, Raili Välimaa, Leena Paakkari

**Affiliations:** ^1^ Research Center for Health Promotion, Faculty of Sport and Health Sciences, University of Jyväskylä, Jyväskylä, Finland; ^2^ Department of Psychology, Faculty of Education and Psychology, University of Jyväskylä, Jyväskylä, Finland

**Keywords:** energy drinks, adolescents, parental monitoring, school achievement, health literacy, educational aspirations

## Abstract

**Objectives:** Energy drink consumption among adolescents has become a notable global phenomenon, and has been associated with numerous negative health outcomes. In order to understand the popularity of energy drinks among adolescents, and to target interventions, it is important to identify the determinants underpinning consumption.

**Methods:** The nationally representative data (cross-sectional) were drawn from the Health Behaviour in School-aged Children (HBSC) surveys, conducted in 2014 and 2018, each comprising 13- and 15-year-old Finnish adolescents (n = 7405).

**Results:** Weekly energy drink consumption increased among Finnish adolescents between 2014 (18.2%) and 2018 (24.4%), especially among girls. In 2018, boys typically consumed more than girls, and 15-year-olds more than 13-year-olds. Moreover, in 2018, weekly energy drink consumption was more prevalent among 15-year-old adolescents with a non-academic educational aspiration (46.0%) than among adolescents with an academic aspiration (18.3%). Gender (boys more than girls), older age (only in 2018), less parental monitoring, lower school achievement, and a lower level of health literacy explained around 28% of the variance in weekly energy drink consumption in both years.

**Conclusion:** According to the findings, interventions to decrease the energy drink consumption, should be targeted at all adolescents, but especially at those with fewer individual resources. The interventions should also pay attention to family-level factors.

## Introduction

Adolescence is a period of rapid physical and psychological changes, and cognitive development [1], within which health-related behaviors begin to form, creating a base for future health [2]. The consumption of caffeine-based and carbonated energy drinks, containing also plant-based stimulants [3] and other ingredients [4], has become a global phenomenon. Their consumption by adolescents has been associated with numerous negative health outcomes, including cardiovascular symptoms [5], psychological symptoms, and headaches [5–7]. National recommendations regarding these beverages have been established in Finland [8], with the aim of limiting their consumption among adolescents. These are in line with European recommendations [9], based on the view that the stimulant content is inappropriate for this target group [10]. In Europe mandatory labeling has been established for products with added caffeine content, indicating that the drinks in question are not recommended for children [11].

Energy drinks are widely consumed by adolescents across the world, and adolescents are the main consumers of these beverages [3]. It is difficult to compare the prevalence of consumption among adolescents internationally and across Europe, due to the different methodologies adopted by studies. In a large European study on annual consumption levels, 68% of adolescents were identified as energy drink consumers on the basis that they had consumed these drinks at least once over the past year [3]. Studies on consumption on a last-three-months basis have produced a figure of 36.4% of adolescents in the United States [12], while within Europe the figures have ranged from 48% (Greece) to 82% (Czech Republic) [3]. The proportion in Finland (65%) has been reported as in line with the average European level [3]. In studies reporting consumption on a monthly basis, figures such as 52.3% have been reported in Europe [13]. Miller et al. [12] found the (at least) weekly energy drink consumption of adolescents in the United States to be 15.1%. A weekly figure of 20.6% was found in Slovakia [14], while in Norway, weekly consumption figures of 15.1–22.4% have been obtained [13].

The available evidence indicates that among adolescents, higher energy drink consumption is linked to male gender [3, 6, 15–17], and older adolescence [13, 15]. It has also been linked to rural residency [13], lower socioeconomic status [13, 18], lower school achievement [17, 19, 20], and lower parental monitoring [21].

More research is needed on the association between energy drink consumption and several other individual resources previously linked to adolescents’ health behaviors. These include health literacy (i.e. a set of competencies that enable individuals to make sound health decisions, and to work on and change the factors affecting their own and others’ health) [22], and educational aspiration (i.e. plans after basic education), both of which have been linked to substance use (e.g. smoking and alcohol consumption) and oral health [23]. As far as we know, there is only limited research on how these might be associated with the consumption of energy drinks.

To understand the energy drink phenomenon among adolescents, it is important to identify the determinants which underpin it. It may then be possible to target intervention at the appropriate group of adolescents, the aim being to intervene at an early stage, prevent health compromising behaviors, and improve health in adolescence, and later in life [2]. Even though the topic has been raised in national political conversations [24], there is a lack of systematic information on the phenomenon in Finland. To gain a deeper understanding of the relevant factors, and of how consumption is evolving, this study aimed to answer the following questions:

1. How prevalent is energy drink consumption, especially on a weekly basis, among Finnish adolescents, and has there been any change in consumption patterns between 2014 and 2018 (RQ1).

2. Which background factors (gender, age, place of residence, geographical region), individual resources (educational aspiration, school achievement, health literacy), and family factors (parental monitoring, family affluence) are associated with weekly energy drink consumption (RQ2), and has there been any change in the associations between 2014 and 2018? (RQ3).

## Methods

### Data

The nationally representative data (cross-sectional) were drawn from the Health Behaviour in School-aged Children (HBSC) surveys among Finnish adolescents. Surveys were conducted in 2014 (a paper-and-pen questionnaire) and 2018 (an online questionnaire via Webropol software, Webropol Oy, Helsinki, Finland) during school hours, anonymously. Participation was voluntary. Stratification in the sampling was carried out according to the European Union NUTS classification (Nomenclature of Territorial Units for Statistics). The sampling was based on the Finnish school register. The participating schools were chosen using a random cluster sampling method adjusted for the province, municipality, and school size (PPS, Proportion Probable Size). The participating classes were randomly selected within each school. In 2014, the study obtained ethical approval according to Finnish national guidelines, and in 2018, from the University of Jyväskylä Ethical Committee. The school principals gave the school-level approval. In 2014, parental/guardian consent was obtained in the form of passive consent, i.e. with the option to withdraw the adolescent from participation. In 2018, some schools required active parental/guardian consent.

The total sample consisted of 7405 Finnish adolescents. In 2014 the sample size was 5195, of which 51% were boys and 49% were girls. The proportion of 13-year-olds was 49%, and the proportion of 15-year-olds was 51%. The sample in 2014 included 184 schools, and the response rate was 86.4%. In 2018 the sample size was 2429, of which 50.1% were boys and 49.9% were girls. The proportion of 13-year-olds was 51.9%, and the proportion of 15-year-olds was 48.1%. The sample in 2018 included 77 schools, and the response rate was 54.1%.

### Measures

The background factors used in the analyses comprised gender (boy/girl), age (13- and 15-year-olds), place of residence (dichotomized as urban/rural), geographical region (covering the South–North dimension, and grouped as Capital city area, Southern Finland, Central Finland, and Northern Finland).

The individual resources examined were educational aspiration, school achievement, and health literacy. Educational aspiration was assessed by asking the students to indicate what they would do after the comprehensive school (at age 15). The response categories were: upper secondary school, vocational school or other vocational training, an apprenticeship, double examination (conducted at both upper secondary school and vocational school), get a job, be unemployed, don’t know. For the analyses, the responses were recoded into two broad categories covering educational aspiration, i.e. academic aspiration (combining general upper secondary school and double examination), and non-academic aspiration (combining vocational school and an apprenticeship). Other responses were not included in the analyses due to their low frequency.

School achievement was measured by asking students to indicate their most recent grade for the first language and for mathematics. The grade for these two subjects were ranked from lowest to highest (grades 4–10) and computed. The mean values of the grades were recoded into three categories, i.e. low (grades 4–6.5), medium (grades 7–8.5), and high (grades 9–10) school achievement.

Health literacy (as a set of competencies to make health decisions and to identify and modify the factors that affect health) was measured by an instrument (HLSAC) created specifically for adolescents [25, 26]. The instrument was computed from 10 different items. Each item began with: “I am confident that...”. The response options were: “not at all true,” “barely true,” “somewhat true,” and “absolutely true.” The responses were computed and recoded as three categories, in line with Paakkari et al. [27]: low (values 10–25), medium (values 26–35), and high (values 36–40) health literacy.

The family factors assessed were family affluence and parental monitoring. The self-reported ‘relative socioeconomic position of the family’ (the family’s material resources, patterns of consumption, and purchasing power) was assessed via the Family Affluence Scale III (FASIII) [28]. FASIII includes six items: ownership of a car, having one’s own bedroom, number of family computers, number of bathrooms, ownership of a dishwasher at home, number of family holidays during the past 12 months. The computed scores were recoded using three categories, in line with the HBSC protocol [29]: low (lowest 20%), medium (middle 60%), and high (highest 20%) family affluence.

Parental monitoring was measured by an instrument of Brown et al. [30], using questions starting with: “How much does your mother/father know about …”. The instrument was computed from five items, relating to who your friends are, how you spend your money, where you are after school, where you go at night, what you do during your free time. The response options were: “don't have or don’t meet,” “doesn’t know anything,” “knows a little,” and “knows a lot.” The mean values were recoded into three categories: low (lowest 33.3%), medium (middle 33.3%), and high (highest 33.3%) parental monitoring.

Energy drink consumption was measured by asking “How often do you eat or drink the following?,” with energy drinks forming one option in a 5-item set. The response options were: “never,” “less than once a week,” “once a week,” “2–4 days a week,” “5–6 days a week,” “once a day, every day,” and “every day, more than once.” The responses were regrouped into three categories: weekly consumption, less than weekly consumption, and no consumption. For the regression analyses, the responses were also dichotomized as two categories, i.e. as weekly and less than weekly/no consumption, in line with the European Food Safety Authority categories [3], and with previous studies [15, 17, 31], on the rationale that weekly energy drink consumption indicates more regular consumption of energy drinks.

### Statistical Analyses

Due to the nested structure of data (with pupils nested in classrooms), descriptive analysis and multilevel mixed-effects binary logistic regression analysis was executed using the Stata (version 16) procedures for complex survey design. The sample design allowed to obtain precise confidence intervals (CIs) for proportions and odds ratios. The variance proportion coefficients (VPCs) were calculated for both age groups via multilevel mixed-effects binary logistic regression analyses, making it possible to measure the school effect. Mplus (version 8.0) with complex methods and the WLSMV estimator was used to obtain the standardized regression coefficients and unbiased standard errors. This method corrects the bias of standard errors due the school effect. Note that because the proportion of Swedish-speaking students in the data was larger than in the general population, all the analyses were weighted accordingly. Moreover, the analyses were weighted for grade in both survey years.

## Results

### Prevalence of Energy Drink Consumption, and Distribution of Consumption by Background Factors, Individual Resources and Family Factors (RQ1)

As shown in the Table 1, weekly energy drink consumption increased among Finnish adolescents between 2014 (18.2%) and 2018 (24.4%). More specifically, the weekly consumption increased among girls (from 9.4% to 16.8%), and among 15-year-olds (from 19.4% to 28.2%). The proportion of adolescents not consuming energy drinks decreased between 2014 (54.8%) and 2018 (49.6%).

**TABLE 1:** The prevalence of energy drink consumption and the distribution of consumption, by background factors, individual resources and family factors (Health Behaviour in School-aged Children, Finland, 2014 and 2018).

	2014					2018				
	Weekly energy drink consumption	Less than weekly energy drink consumption	No energy drink consumption	Total % (*n*)	2014 *p* value	Weekly energy drink consumption	Less than weekly energy drink consumption	No energy drink consumption	Total % (*n*)	2018 *p* value	2014 vs. 2018 *p* value
Gender					<0.001					<0.001	
Boys	27.2 [24.8–29.7]	32.3 [30.1–34.6]	40.5 [37.9–43.2]	100 (2460)		32.1 [28.7–35.7]	27.5 [24.8–30.5]	40.4 [36.7–44.1]	100 (1203)		0.02
Girls	9.4 [8.1–10.9]	21.8 [19.8–23.9]	68.9 [66.4–71.2]	100 (2496)		16.8 [14.2–19.7]	24.4 [21.2–27.9]	58.9 [54.5–63.1]	100 (1203)		<0.001
All	18.2 [16.7–19.8]	27.0 [25.4–28.6]	54.8 [52.8–56.8]	100 (4956)		24.4 [22.2–26.7]	26.0 [23.8–28.2]	49.6 [46.7–52.6]	100 (2406)		<0.001
Age					<0.01					<0.001	
13-year-olds	17.0 [14.9–19.4]	24.7 [22.5–27.1]	58.3 [55.4–61.1]	100 (2487)		20.6 [17.5–24.0]	23.2 [20.6–26.1]	56.2 [51.8–60.6]	100 (1239)		0.16
15-year-olds	19.4 [17.3–21.6]	29.2 [27.0–31.5]	51.4 [48.6–51.2]	100 (2469)		28.2 [25.1–31.5]	28.7 [26.0–31.6]	43.1 [39.8–46.5]	100 (1167)		<0.001
Family affluence					0.28					0.28	
Low	19.6 [16.5–23.2]	28.9 [25.2–33.0]	51.5 [47.0–55.9]	100 (561)		26.1 [21.3–31.7]	26.6 [21.6–32.3]	47.3 [41.2–53.4]	100 (229)		0.12
Medium	17.6 [15.9–19.4]	27.5 [25.5–29.6]	54.9 [52.6–57.2]	100 (2701)		25.0 [22.1–28.0]	25.6 [22.7–28.8]	49.4 [45.6–53.3]	100 (1218)		<0.001
High	17.5 [15.2–20.1]	25.5 [23.0–28.3]	57.0 [53.5–60.3]	100 (1515)		21.1 [17.9–24.6]	27.1 [23.9–30.5]	51.9 [47.8–55.9]	100 (842)		0.10
Parental monitoring					<0.001					<0.001	
Low	29.4 [26.5–32.5]	32.6 [29.8–35.5]	38.0 [35.1–40.9]	100 (1489)		41.1 [36.2–46.3]	28.8 [25.0–32.9]	30.1 [25.8–34.8]	100 (674)		<0.001
Medium	13.8 [12.2–15.6]	27.9 [25.7–30.2]	58.3 [55.8–60.7]	100 (1939)		18.6 [15.7–21.9]	27.0 [23.4–30.9]	54.4 [49.3–59.3]	100 (838)		0.04
High	11.0 [9.2–13.1]	20.4 [18.0–22.9]	68.6 [65.6–71.6]	100 (1456)		13.0 [10.7–15.7]	23.2 [20.5–26.1]	63.8 [60.2–67.3]	100 (804)		0.11
Place of residence					0.47					0.98	
Urban	17.8 [16.0–19.8]	26.5 [24.6–28.6]	55.7 [53.1–58.3]	100 (2978)		24.5 [21.8–27.5]	25.7 [22.9–28.7]	49.8 [46.2–53.3]	100 (1254)		<0.001
Rural	18.7 [16.4–21.2]	27.9 [25.4–30.6]	53.4 [50.3–56.4]	100 (1942)		24.3 [21.7–27.9]	26.2 [23.1–29.5]	49.5 [45.0–54.0]	100 (1145)		0.03
Geographical region					<0.01					0.03	
Capital city area	14.5 [10.1–20.4]	20.5 [16.3–25.4]	65.0 [58.0–71.5]	100 (711)		20.9 [15.3–27.9]	19.8 [14.3–26.7]	59.3 [51.0–67.1]	100 (248)		0.25
Southern Finland	18.5 [16.1–21.1]	25.6 [23.3–28.1]	55.9 [52.6–59.1]	100 (1674)		23.8 [21.1–26.6]	26.6 [23.4–30.1]	49.6 [45.0–54.3]	100 (1077)		0.02
Central Finland	20.0 [17.6–22.5]	28.6 [25.9–31.4]	51.5 [48.4–54.6]	100 (1785)		24.4 [19.8–29.8]	26.8 [23.2–30.7]	48.8 [44.0–53.6]	100 (784)		0.20
Northern Finland	16.9 [13.1–21.6]	31.6 [27.5–36.0]	51.5 [46.6–56.4]	100 (703)		31.0 [25.5–37.1]	28.1 [23.0–33.7]	40.9 [36.6–45.4]	100 (276)		<0.001
Educational aspiration (15-year-olds)					<0.001					<0.001	
Academic	11.1 [9.8–12.5]	24.0 [22.1–26.0]	64.9 [62.6–67.1]	100 (2997)		18.3 [15.5–21.4]	30.3 [27.0–33.9]	51.4 [47.3–55.4]	100 (746)		<0.001
Non-academic	30.6 [27.6–33.8]	34.0 [31.4–36.8]	35.4 [32.5–38.4]	100 (1501)		46.0 [40.8–51.4]	26.2 [21.9–30.9]	27.8 [23.4–32.7]	100 (389)		<0.001
School achievement					<0.001					<0.001	
Low	38.9 [34.8–43.2]	31.9 [28.3–35.7]	29.3 [25.7–33.2]	100 (733)		53.4 [48.4–58.4]	24.3 [20.3–28.8]	22.3 [18.3–26.8]	100 (368)		<0.001
Medium	18.4 [16.6–20.3]	29.0 [27.0–31.0]	52.7 [50.4–54.9]	100 (2963)		23.1 [20.5–26.0]	29.0 [26.5–31.6]	47.9 [44.5–51.3]	100 (1346)		<0.01
High	5.6 [4.3–7.3]	19.2 [16.9–21.9]	75.2 [72.2–78.0]	100 (1225)		11.0 [8.6–14.1]	21.0 [17.2–25.5]	67.9 [63.1–72.4]	100 (628)		<0.01
Health literacy					<0.001					<0.001	
Low	32.4 [27.8–37.4]	30.5 [26.1–35.3]	37.1 [32.0–42.5]	100 (431)		42.9 [36.2–49.7]	25.3 [19.0–32.8]	31.9 [25.1–39.5]	100 (208)		0.07
Medium	17.6 [15.8–19.4]	28.9 [26.9–31.0]	53.5 [51.1–55.9]	100 (2650)		24.4 [21.2–27.8]	26.2 [23.5–29.2]	49.5 [45.4–53.3]	100 (1239)		<0.001
High	12.8 [11.0–14.8]	23.2 [21.0–25.8]	64.0 [61.1–66.8]	100 (1552)		17.3 [14.9–20.1]	26.7 [23.4–30.2]	56.0 [52.2–59.7]	100 (783)		<0.01

In 2018, half of the adolescents reported consumption of energy drinks (combining weekly and less than weekly consumption). A quarter of the adolescents reported consumption of energy drinks on a weekly basis, boys (32.1%) more typically than girls (16.8%), and 15-year-olds (28.2%) more typically than 13-year-olds (20.6%). Less than weekly consumption was as prevalent as weekly consumption among 13- and 15-year-olds, and among boys, but not among girls. Half of the adolescents did not consume energy drinks at all. The proportion of adolescents not consuming energy drinks was higher among girls (58.9%) than boys (40.4%), and among 13-year-olds (56.2%) than 15-year-olds (43.1%).

Table 1 indicates that in both survey years, weekly energy drink consumption was more prevalent when scores for parental monitoring, school achievement, and health literacy were low. As regards geographical regions, the increase in energy drink consumption between 2014 and 2018 was statistically significant only in Southern Finland (from 18.5% to 23.8%) and in Northern Finland (from 16.9% to 31.0%). Furthermore, in both survey years, weekly energy drink consumption was more prevalent among adolescents with a non-academic educational aspiration (increasing from 30.6% to 46.0%) than among adolescents with an academic aspiration (increasing from 11.1% to 18.3%).

### Associations Between Weekly Energy Drink Consumption and Background Factors, Individual Resources and Family Factors (RQ2) in 2014 and 2018 (RQ3)

Among 13-year-olds, weekly energy drink consumers were more likely to be a boy, live in Central Finland, report medium or low parental monitoring, report medium or low school achievement, and report low health literacy (Table 2). Among 13-year-olds, the inter-school variance (variance partition coefficient, VPC) was 11.8% of the total variance.

**TABLE 2:** Multilevel mixed-effects binary logistic regression on the associations between weekly energy drink consumption and background factors, individual resources and family factors, among 13- and 15-year-olds (Health Behaviour in School-aged Children, Finland, 2014 and 2018).

		Model 1 (13-year-olds^a^) *n* = 3217			Model 2 (15-year-olds^b^) *n* = 3197		
		OR	95% CI	*p*	OR	95% CI	*p*
Gender	Girl	1.00			1.00	
	Boy	2.39	[1.91–2.98]	<0.001	2.71	[2.21–3.32]	<0.001
Survey year	2014	1.00			1.00		
	2018	1.54	[1.13–2.09]	<0.01	1.93	[1.56–2.39]	<0.001
Place of residence	Urban	1.00			1.00		
	Rural	1.04	[0.81–1.33]	0.79	0.99	[0.81–1.24]	0.99
Geographical region	Capital city area	1.00			1.00		
	Southern Finland	1.39	[0.87–2.23]	0.17	0.85	[0.60–1.21]	0.38
	Central Finland	1.67	[1.04–2.68]	0.04	1.12	[0.78–1.61]	0.54
	Northern Finland	1.71	[0.98–2.98]	0.06	1.08	[0.70–1.65]	0.74
Family affluence	High	1.00			1.00		
	Medium	0.83	[0.65–1.05]	0.11	0.79	[0.64–0.98]	0.04
	Low	0.85	[0.59–1.23]	0.40	0.72	[0.51–1.02]	0.07
Parental monitoring	High	1.00			1.00		
	Medium	1.65	[1.22–2.22]	<0.01	1.26	[0.98–1.63]	0.07
	Low	5.71	[4.25–7.67]	<0.001	2.24	[1.75–2.88]	<0.001
School achievement	High	1.00			1.00		
	Medium	2.71	[1.96–3.75]	<0.001	2.57	[1.87–3.53]	<0.001
	Low	5.46	[3.66–8.16]	<0.001	4.58	[3.13–6.71]	<0.001
Health literacy	High	1.00			1.00		
	Medium	1.07	[0.82–1.39]	0.63	1.26	[1.01–1.56]	0.04
	Low	1.75	[1.22–2.49]	<0.01	1.52	[1.07–2.16]	0.02
Educational aspiration^c^	Academic				1.00		
	Non-academic				1.88	[1.50–2.36]	<0.001

OR, odds ratio; CI, confidence interval.

^a^ School effect *p*<0.001, variance proportion coefficient: 0.118.

^b^ School effect *p*=0.12, variance proportion coefficient: 0.0169.

^c^ In both 2014 and 2018, educational aspiration was included in the survey only for 15-year-olds.

Among 15-year-olds, weekly energy drink consumers were more likely to be a boy, report low parental monitoring, report medium or low school achievement, report medium or low health literacy, and report non-academic aspiration. By contrast, adolescents reporting medium family affluence were less likely to consume energy drinks on a weekly basis.

These associations were further confirmed via the linear regression model (Table 3). On the basis of the results of logistic regression analysis, gender, age, parental monitoring, school achievement, and health literacy were chosen to predict the weekly energy drink consumption. Educational aspiration was not included in the model, in order to include both age groups in the model.

**TABLE 3:** Linear regression model on the strength of the associations between weekly energy drink consumption and gender, age, parental monitoring, school achievement, and health literacy, separately by year (Health Behaviour in School-aged Children, Finland, 2014 and 2018).

	2014			2018		
	*R* ^2^ = 0.28, *p* value <0.001 (*n* = 4592)			*R* ^2^ = 0.28, *p* value <0.001 (*n* = 2170)		
	β	95% CI	*p* value	β	95% CI	*p* value
Gender	−0.256^a^	[−0.302 ‐ −0.211]	<0.001	−0.145^a^	[−0.212 ‐ −0.078]	<0.001
Age	0.019^b^	[−0.032 ‐ 0.071]	0.47	0.108^b^	[0.044 ‐ 0.171]	<0.01
Parental monitoring	−0.243^c^	[−0.287 ‐ −0.198]	<0.001	−0.258^c^	[−0.321 ‐ −0.196]	<0.001
School achievement	−0.249^d^	[−0.291 ‐ −0.207]	<0.001	−0.286^d^	[−0.348 ‐ −0.225]	<0.001
Health literacy	−0.071^e^	[−0.110 ‐ −0.033]	<0.001	−0.111^e^	[−0.161 ‐ −0.062]	<0.001

a For girls.

^b^ For 15-year-olds.

^c-e^ The greater the value below zero, the higher the level of the selected variable.

Overall, the chosen factors showed medium effect sizes [32], explaining 28% of weekly energy drink consumption in 2014 and in 2018. When all the factors were analyzed simultaneously, gender (boys more than girls), less parental monitoring, lower school achievement, and a lower level of health literacy, explained the variance in weekly energy drink consumption in both years. Age (greater consumption among 15-year-olds than 13-year-olds) explained the variance only in 2018.

According to the confidence intervals of the regression coefficients, gender, parental monitoring, school achievement, and health literacy were equally strongly associated with weekly energy drink consumption in both 2014 and 2018.

## Discussion

According to our study, in 2018 every second Finnish adolescent reported consumption of energy drinks, and every fourth adolescent reported consumption on a weekly basis. Variation emerged according to background factors, individual resources, and family factors. Weekly consumption increased from 2014 to 2018, suggesting that energy drinks have not lost their popularity among Finnish adolescents, despite the fact that these beverages are inappropriate for this target group [9–11]. It should nevertheless be noted that the increase in consumption is inconsistent with some recent international findings [33]. Our results indicate that in 2018 energy drink consumption was more common among older adolescents, confirming previous findings [13, 15]. Longitudinal research is needed to explore, whether the consumption of these beverages is merely an experimental or passing phase in adolescence, or rather a phenomenon that increases during adolescence.

Energy drink consumption and the advertising related to it, has traditionally been linked to masculinity norms [12]. However, more recent evidence has indicated that the marketing of these beverages has changed, and that adolescents themselves have the impression that specific drinks are targeted at either male or female consumers [34]. The selection of energy drinks has increased, with options differing by package and overall image. Indeed, as our results indicate, girls’ weekly consumption has increased considerably, raising the possibility that the gender normative trend of specific health behaviors has started to change. This despite the fact that boys consume energy drinks more than girls [3, 6, 15–17], a finding confirmed also by this study. The popularity of these drinks among boys, has been explained via the fact that energy drink consumption is linked to screen time [35] and to gaming [31], both of which are higher among boys.

Health behaviors track forward to adulthood, calling for a deeper understanding not only of the individuals’ background factors reflected above, but also of individual resources, and of family factors, as these all affect this specific health behavior. It has been reported that adolescents have limited knowledge of the ingredients of energy drinks, especially of their key ingredient, i.e. caffeine [16, 36], and that they tend to confuse them with sports drinks [36] or with other soft drinks [16, 31, 36]. Moreover, weekly energy drink consumption has been associated with adolescents’ perceptions that energy drinks are safe drinks for teens [37]. As our study indicates, weekly energy drink consumption was associated with low and medium health literacy, confirming the need to undertake targeted interventions and to develop adolescents’ knowledge and skills related to energy drinks.

Besides lower levels of health literacy, the weekly consumption of energy drinks was found to be more likely among adolescents with lower academic achievement, consistent with the notion that current school achievement is an important indicator of the level of health and health behavior (see also [17, 19, 20]). Moreover, our study indicated that weekly energy drink consumption was more prevalent among adolescents with non-academic educational plans. There is clear evidence on health and health behavior differences between persons with academic and non-academic aspirations, providing a rough indication of adolescents’ future social position and health inequalities [38]. It has been reported that students following the non-academic path have more health compromising behaviors than students following the academic path [38, 39]. Within Finland, students in vocational schools consume energy drinks to a considerably greater extent than students in general upper secondary schools [40].

Interestingly, family affluence, which is typically seen as a strong predictor of health behaviors [41], had no significant role in energy drink consumption in the sample as a whole; nevertheless, a weak association was found when age-specific consumption was examined. Among 15-year-olds, those reporting medium level family affluence were less likely to consume energy drinks weekly than those reporting high family affluence. This contrasts with the study by Degirmenci et al. [13] who found that lower socioeconomic status is associated with higher energy drink consumption. At the same time, it has been reported that higher parental monitoring is associated with a lowered risk of health compromising behaviors [42] including energy drink consumption [21]. In line with this, we found that the more parents knew about their child’s free time activities, their company, and their money expenditure, the less likely it was that adolescent consumed energy drinks weekly – a finding underlining the role of parents in the consumption of these drinks.

In the lives of adolescents, an important source of information on energy drinks could potentially be family members, who may well consume energy drinks themselves [36]. It is therefore important also to ensure that information reaches the home, so that parents have a good level of knowledge on energy drink constituents and on their possible effects among adolescents. In some existing studies, parents’ actions have actually been seen to increase energy drink consumption, insofar as they purchase them for the adolescents and encourage adolescents to drink them, in order to decrease fatigue or improve sports performance [36].

Our findings indicate that in seeking to decrease weekly energy drink consumption, certain factors should be examined in more detail. It was notable that factors such as gender, age (only in 2018), school achievement, parental monitoring, and health literacy explained almost 30% of the variation in weekly energy drink consumption, in both 2014 and 2018. The percentages are striking, given that (apart from gender) several of these factors are modifiable. It seems that if one is aiming to influence health disparities among adolescents, interventions should be targeted at all adolescents, but especially at those with fewer individual resources. In addition, the interventions should pay attention to family-level factors.

Among the strengths of the current study, one can number valid measures, a large, nationally representative database, and two separate survey years. The study provided important information on energy drink consumption in Finland. However, due to the cross-sectional study design, it is not possible to make causal inferences between consumption and the measured individual determinants. In addition, the survey relied on self-reports, so assessment of energy drink consumption was subject to self-reporting bias. The current survey did not include the precise quantity of energy drinks consumed over a certain period of time; hence, the quantity of energy drinks consumed at a given time remains unclear. Note also that in this study energy drink consumption was studied separately from other habits. Further research is needed to examine energy consumption together with other health habits and contextual factors (e.g. peers and family) to build a coherent understanding of the phenomenon.

## Conclusion

Energy drink consumption, especially on a weekly basis, increased among Finnish adolescents between 2014 and 2018, and among girls in particular. The findings suggest that special attention should be paid to those adolescents who aim at a non-academic education, or who have lower school achievement or a lower level of health literacy. It is important to understand the significant role that school and health education could play in decreasing the consumption of energy drinks, for instance by developing the health literacy of adolescents. Furthermore, we suggest that parental monitoring of friends, expenditure, and free time activities may be important as a preventive factor.

## Data Availability

The data analyzed in this study is subject to the following licenses/restrictions: **The HBSC Data Management Centre distributes data in accordance with the HBSC data access policy**. Requests to access these datasets should be directed to dmc@hbsc.org.
